# GluA2-Containing AMPA Receptors Distinguish Ribbon-Associated from Ribbonless Afferent Contacts on Rat Cochlear Hair Cells^1^,^2^,^3^

**DOI:** 10.1523/ENEURO.0078-16.2016

**Published:** 2016-05-12

**Authors:** Rodrigo Martinez-Monedero, Chang Liu, Catherine Weisz, Pankhuri Vyas, Paul Albert Fuchs, Elisabeth Glowatzki

**Affiliations:** 1 Department of Otolaryngology-Head and Neck Surgery, the Center for Hearing and Balance; 2 Department of Neuroscience; 3 The Center for Sensory Biology, Institute for Basic Biomedical Sciences, The Johns Hopkins University School of Medicine, Baltimore, Maryland 21205

**Keywords:** AMPA receptors, cochlear afferents, outer hair cells, postsynaptic densities

## Abstract

Mechanosensory hair cells release glutamate at ribbon synapses to excite postsynaptic afferent neurons, via AMPA-type ionotropic glutamate receptors (AMPARs). However, type II afferent neurons contacting outer hair cells in the mammalian cochlea were thought to differ in this respect, failing to show GluA immunolabeling and with many “ribbonless” afferent contacts. Here it is shown that antibodies to the AMPAR subunit GluA2 labeled afferent contacts below inner and outer hair cells in the rat cochlea, and that synaptic currents in type II afferents had AMPAR-specific pharmacology. Only half the postsynaptic densities of type II afferents that labeled for PSD-95, Shank, or Homer were associated with GluA2 immunopuncta or presynaptic ribbons, the “empty slots” corresponding to ribbonless contacts described previously. These results extend the universality of AMPAergic transmission by hair cells, and support the existence of silent afferent contacts.

## Significance Statement

Transmission from cochlear hair cells to afferent neurons is mediated by ionotropic glutamate receptors. Inner hair cells efficiently drive acoustic coding in type I afferents that express AMPA-type ionotropic glutamate [glutamate receptor A2 (GluA2)]-containing AMPA receptors. Type II cochlear afferents differ from type I afferents not only in number, caliber, myelination, and excitability, but also in the utility of their terminal contacts with hair cells. Outer hair cell to type II afferent transmission is far less effective, but also uses GluA2-containing receptors. Only half the type II afferent boutons that immunolabeled for postsynaptic density proteins had GluA2 receptors. And, only GluA2-containing contacts were associated with presynaptic ribbons, the active zones for the release of the hair cell transmitter.

## Introduction

Mechanosensory hair cells of vertebrates release glutamate (Glu) to excite afferent neurons. This holds true as well within the mammalian cochlea, where acoustic information is transmitted from inner hair cells (IHCs) to the predominant (95%) myelinated type I afferents by the activation of AMPA receptors (AMPARs; Ruel et al., 2000; Glowatzki and Fuchs, 2002). The fewer, smaller-caliber, unmyelinated type II afferents contact many outer hair cells (OHCs), but are only weakly activated by glutamate release at those connections (Weisz et al., 2012). Physiological studies suggest that AMPARs also mediate OHC transmission to type II afferents (Weisz et al., 2009), although this result runs counter to the conclusion based on immunolabeling that OHC synapses are not AMPAR dependent (Ottersen et al., 1998; Matsubara et al., 1999; Liberman et al., 2011). Synaptic signaling from OHCs to type II afferents is further complicated by the presence of “ribbonless” contacts (Dunn and Morest, 1975; Liberman et al., 1990), and by the expression of kainate receptors at both ribbon-containing and ribbonless contacts (Fujikawa et al., 2014). Given these discrepancies and unknowns, it was of interest to explore further the pharmacology of postsynaptic currents, to characterize the distribution of postsynaptic proteins at the OHC to type II afferent contacts, and to compare these with the better characterized IHC-to-type I afferent contact.

In mammals, heteromeric AMPA receptors are made of the following four different subunits: GluA1-4, encoded by separate but related genes (Köhler et al., 1994; Borges and Dingledine, 1998). Previous attempts with antibodies to the glutamate receptor A2/3 (GluA2/3) heteromer failed to label type II contacts onto OHCs (Matsubara et al., 1996; Knipper et al., 1997; Eybalin et al., 2004; Meyer et al., 2009; Liberman et al., 2011). More recently, antibodies specific for the GluA2 subunit showed specific labeling beneath IHCs, but not OHCs, of adult mice (Fujikawa et al., 2014; Fernandez et al., 2015; Liberman et al., 2015). In contrast, the present work found GluA2 immunoreactivity in association with ribbon synapses of inner and outer hair cells of adult and young rats. Consistent with that result, glutamatergic synaptic currents in type II afferents of young rats were blocked by the AMPAR-specific antagonist CP-465,022 (Lazzaro et al., 2002; Balannik et al., 2005).

The organization of afferent synapses also was analyzed by immunolabel against postsynaptic density proteins. PSD-95, a PDZ domain-containing protein of the MAGUK (membrane-associated guanylate kinase) family, is a ubiquitous component of glutamatergic synapses, including those of cochlear hair cells (Davies et al., 2001). Shank proteins interconnect many components of the postsynaptic density with the cytoskeletal matrix (Sheng and Kim, 2000), including NMDA, AMPA, and metabotropic glutamate receptors (mGluRs), the last of which is connected through the coupling protein Homer (Boeckers, 2006). In the present work, antibodies to PSD-95, Shank, and Homer were used to map their distribution under IHCs and OHCs. Consistent with previous reports (Meyer et al., 2009; Liberman et al., 2011; Wang and Green, 2011), the majority of postsynaptic densities in type I afferents beneath IHCs were associated with GluA-positive immunopuncta and presynaptic ribbons. Beneath OHCs, however, only GluA2-containing postsynaptic densities (about half the total) were juxtaposed to presynaptic ribbons. The GluA2-lacking and ribbon-lacking postsynaptic densities are reminiscent of ribbonless afferent contacts that have been described by others (Dunn and Morest, 1975; Nadol, 1983; Liberman et al., 1990) and may indicate that type II afferents possess a type of “silent synapse” that could be activated in response to changing conditions.

## Materials and Methods

Adult or 1- to 2-week-old Sprague Dawley albino rats of either sex (CD IGS rats, Charles River Laboratories) were deeply anesthetized by isoflurane inhalation and decapitated, and the temporal bone was quickly removed. All experimental procedures involving animals were approved by Johns Hopkins University Animal Care and Use Committee.

### Immunohistochemistry

After removing the inner ear from the skull, a small hole was made in the apical bone of the cochlea to allow flow of the solution. For fixation of the tissue, 4% paraformaldehyde (Electron Microscopy Sciences) prepared in PBS (1× PBS), pH 7.4, was perfused through the round and oval windows into the cochlea, and the tissue was kept in fixative for 30 min to 2 h at 4°C. After three washes in PBS, cochlear tissue was microdissected and freed from bone to facilitate better access of the antibodies to the tissue. In a typical experiment, cochlear tissue from two ears, three pieces per ear, were processed together. The tissue pieces were transferred with a spoon into a drop of PBS located in the center region (∼1 cm in diameter) of a microscope slide. Next, whole-mount preparations were incubated in a permeabilizing solution with 0.5% of NP40 detergent in PBS for 10–60 min at 4°C. Tissue was exposed to 1% bovine serum albumin (BSA) and 10% heat-inactivated goat serum in PBS for 1 h at room temperature (RT) to reduce nonspecific labeling. Primary antibodies were applied overnight at 4°C in 5% heat-inactivated goat serum and 1% BSA with or without 0.5% NP40 detergent, depending on the antibody used.

The following primary antibodies were used: GluA2 monoclonal mouse antibody (catalog #MAB397, Chemicon); GluA1 polyclonal rabbit antibody, GluA2N polyclonal rabbit antibody, GluA3N polyclonal rabbit antibody, and GluA4N polyclonal rabbit antibody (R. Huganir, The Johns Hopkins University School of Medicine, Baltimore, MD; Araki et al., 2010); CtBP2 rabbit antibody (catalog #BS2287, BioWorld Tech); CtBP2 mouse antibody (catalog #612044, BD Biosciences); Shank and Homer1 polyclonal antibodies (P. Worley, The Johns Hopkins University School of Medicine); PSD-95 polyclonal (catalog #610495, BD Bioscience); and PSD-95 monoclonal (catalog #73/028, UC Davis/NIH NeuroMab Facility). After overnight incubation with primary antibodies, samples were washed and incubated for 1 h at room temperature with the secondary antibodies. Alexa Fluor 488 goat anti-rabbit and Alexa Fluor 568 goat anti-mouse (Invitrogen), centrifuged at high speed and diluted at 1:1000 in 1× PBS, were used as secondary antibodies. Samples were rinsed three times for 10 min each in PBS at RT before mounting and viewing.

### Controls

The specificity of GluA2 antibodies was confirmed in control experiments on GluA2-null mice. GluA2-null heterozygotes (Huganir Laboratory, Solomon H. Snyder Department of Neuroscience, Johns Hopkins University School of Medicine) were bred to provide homozygous-null mice; genotype was confirmed by PCR. Tissue was fixed and immunolabeled using the same procedure as for experimental animals. In three GluA2-null mice, there was good labeling of presynaptic ribbons with the antibody against CtBP2 in both OHCs and IHCs, but no GluA2 label. Wild-type littermates had positive CtBP2 and GluA2 labels in the IHC area.

### Secondary antibody controls

This work was designed to address synaptic structure and location at the cellular level, rather than the submicrometer distribution of each protein component. Thus, simultaneous labeling with presynaptic and postsynaptic markers could present significant overlap in *z*-scan confocal microscopy and was the desired goal of this work; to evaluate the regional colocalization of a variety of postsynaptic proteins. However, this raised the question of whether such overlap represents true colocalization or results from cross talk between channels for the different fluorophores (Alexa Fluor 488 and 568) that were conjugated to secondary antibodies. Thus, some samples were incubated with only one primary antibody (e.g., CtBP2 antibody without GluA2 antibody; GluA2 antibody without CtBP2 antibody) followed by incubation with both secondary antibodies presented together. In neither case was there a colocalized signal for the absent primary antibody.

### Intracellular recording from type II cochlear afferents

The apical turn of the cochlea was dissected from young rats [postnatal day 7 (P7) to P9], followed by the removal of stria vascularis and tectorial membrane. The cochlear turn was then secured onto a coverslip by an insect pin serving as a spring clamp and imaged under a microscope (Examiner D1, Carl Zeiss) using a 40× water-immersion objective and a camera with contrast enhancement (model C2400-62, Hamamatsu). Three to four OHCs were removed with a glass suction pipette to expose the dendrites of type II cochlear afferents. The extracellular solution contained the following (in mm): 5.8 KCl, 144 NaCl, 1.3 CaCl_2_, 0.9 MgCl_2_, 0.7 NaH_2_PO4, 5 glucose, and 10 HEPES, pH 7.4. Giga-ohm seal pipettes were pulled from 1 mm borosilicate glass (WPI) with a final pipette resistance of 7-9 MΩ after fire polishing, and were filled with an intracellular solution containing the following (in mm): 110 K-methane sulfonate, 20 KCl, 0.1 CaCl_2_, 3.5 MgCl_2_, 5 K-EGTA, 5 HEPES, 5 Na_2_phosphocreatine, and 0.3 Tris-GTP, pH 7.2. Junction potentials (10 mV) for this solution were corrected in the reported membrane potential. The series resistance was <30 MΩ (membrane test of the pCLAMP 10.3 software, Molecular Devices) and was not corrected for the small currents recorded here. Intracellular recording from type II afferents was confirmed by the characteristic voltage-gated currents elicited by a series of voltage steps and the presence of rapid synaptic currents. The frequency of “spontaneous” synaptic currents was increased by bathing the tissue in a 40 mm potassium saline solution (substituted for sodium) to depolarize hair cells. Synaptic events were collected for at least 1 min prior to the addition of the AMPAR antagonist CP-465,022 to the high potassium perfusate via a large-bore application pipette positioned close to the recording site. All reagents were obtained from Sigma-Aldrich, except for philanthotoxin and CP-465,022, which were obtained from Tocris Bioscience.

Recordings (at room temperature) were made with a MultiClamp 700B amplifier and a Digidata 1440A Digitizer (Molecular Devices), which were controlled by pCLAMP 10.3 software (Molecular Devices), sampled at 25 kHz, and low-pass filtered at 1–10 kHz. Data were analyzed in Clampex (Molecular Devices) and Origin 9.0 (OriginLab). EPSCs were selected and analyzed using MiniAnalysis software (Synaptosoft) with an amplitude criterion three times the root mean square of the noise.

### Type II fiber biocytin filling and peroxidase reaction for immunohistochemistry and detection of labeling

The 0.3% biocytin (3.0 mg/ml) was added to the intracellular solution of the patch pipettes for delivery into type II afferents via whole-fiber tight-seal recordings. The tracer was detected *post hoc* using streptavidin-conjugated horseradish peroxidase, or streptavidin-conjugated fluorescent labeling. In some experiments, the cochlear tissue was preloaded (30 s, room temperature) with 5 µm FM1-43FX (Invitrogen), a fluorescent dye that rapidly enters through mechanotransduction channels and partitions into the hair cell membrane. Cochlear explants with filled type II afferents were fixed immediately after recordings in 4% paraformaldehyde (v/v) overnight at 4°C. After washing in PBS, the tissue was quenched in 10% H_2_O_2_ (in 10% methanol and 90% PBS) for 10 min, then permeabilized in 2% Triton in PBS for 1 h at room temperature. Avidin–biotin complex (Vectastain ABC Kit, Vector Laboratories) was added, and the tissue was incubated overnight at 4°C. Under a dissection microscope (model MS5, Leica), each individual tissue was reacted with a diaminobenzidine-based peroxide substrate (ImmPACT DAB, Vector Laboratories) for ∼10 min, until the cell and its arborization were visible. The tissue was then transferred and mounted onto a microscope slide.

A second set of experiments combined fluorescent labeling of the fiber (biocytin, streptavidin Alexa Fluor 488) with immunofluorescent labeling of OHCs. The tissue with the filled type II afferent fiber was fixed in 4% PFA for 10–60 min at 4°C. Then the tissue was exposed to 1% BSA and 10% heat-inactivated goat serum in PBS for 1 h at RT to reduce nonspecific labeling. Streptavidin-Alexa Fluor 488 conjugate and CtBP2 or PSD-95 antibodies were applied overnight at 4°C in 5% heat-inactivated goat serum and 1% BSA. Samples were washed and incubated for 1 h at RT with the secondary antibodies Alexa Fluor 568 goat anti-rabbit and Alexa Fluor 633 goat anti-mouse (Invitrogen). Secondary antibodies were centrifuged at high speed and diluted at 1:1000 in 1× PBS before use. Samples were rinsed three times for 10 min each in PBS at RT before mounting and viewing.

### Image acquisition

Mounted cochlear turns were imaged using a confocal laser-scanning microscope (LSM 510 Meta, Zeiss) with appropriate excitation and emission filters. A Plan-Apochromat 100× oil-objective with a numerical aperture of 1.4 was used. Whole-mount preparations of the apex-middle region of the adult (>2 months old) rat cochlea were used unless otherwise specified. For every experimental condition, cochlear turns of rats from at least three different litters were analyzed. From every organ of Corti, *z*-stack projections were taken from at least three areas in the lower apex-upper middle turn of the organ of Corti. Analysis was focused on the endings of the type I and type II spiral ganglion afferent fibers that innervate IHCs and OHCs, respectively, and each stack contained the entire synaptic pole of the hair cells as viewed from the endolymphatic surface of the organ of Corti. Each acquisition frame covered ∼24 OHCs and ∼5 IHCs (visualized by CtBP2 immunoreactivity in nuclei or by background fluorescence in other experiments). The confocal detection volumes of all image channels were equalized simultaneously. Images were taken with sequential scanning with multitrack acquisition to reduce cross talk. Care was taken to minimize pixel saturation in each image stack. For morphological analysis, stacks of confocal images (0.37 µm maximum *z*-intervals) were imported into Imaris XT software 7.4 (with image acquisition using the LSM 510 Meta microscope) for 3-D reconstruction and quantification.

### 3-D morphometry, puncta quantification, and juxtaposition

All quantitative analysis was performed with Imaris XT software (version 7.4) using raw image stacks, without any deconvolution, filtering, gamma correction, or resampling. Antibody labeling occurred in discrete patches or in puncta at the base of the hair cells. The total number of synaptic markers was counted in each stack and divided by the number of hair cells. To evaluate the juxtapositions among CtBP2, GluA2, PSD-95, Shank, and Homer immunolabeling, an iso-surface of each signal was created in independent color channels. Puncta volumes were computed using functions that provide 3-D rendering and visualization of iso-surfaces enveloping all pixel clusters with intensities greater than a user-defined criterion value (and with greater than a minimum number of enveloped pixels). Puncta volumes were computed along the *x*-, *y*-, and *z*-coordinates of their centers. Surface-to-surface measurements were used to create a distance transformation channel with an intensity minimum representing the closest distance between two objects. A threshold was set at ≤0.5 µm to define the juxtaposition of two different puncta. The computed results were corroborated by visual inspection of the puncta. Significance was measured using the Student’s *t* test or one-way ANOVA followed by Bonferroni’s multiple comparison test. All data are reported as the mean ± SEM, unless otherwise noted. GraphPad Prism4 was used to compute the statistical results.

## Results

### Relationship of presynaptic ribbons and postsynaptic GluA2 clusters at IHC and OHC afferent contacts

**Figure 1.**
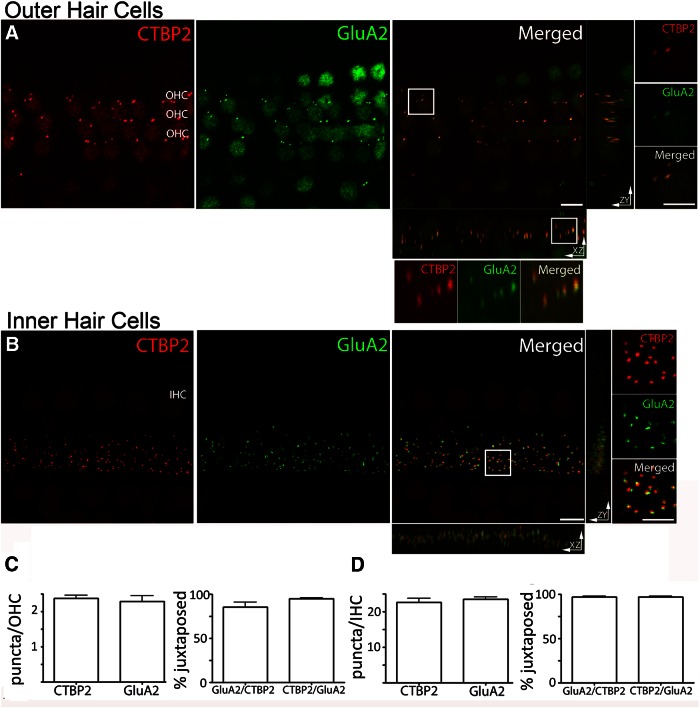
Ribbons and AMPAR clusters in cochlear whole mounts, and maximum intensity projections of confocal *z*-stacks of the medial region of the organ of Corti from an adult rat viewed from the endolymphatic surface including 24 adjacent OHCs and 5 IHCs. ***A***, OHCs: immunolabel for the presynaptic ribbon marker (CtBP2, red channel). Immunolabel for the postsynaptic marker GluA2 (green channel). Merged and magnified inserts: CtBP2 and GluA2 puncta overlapped in the *x*- to *y*-plane. Rotation to the *z*- to *x*-planes or *z*- to *y*-planes reveals displacement between presynaptic and postsynaptic markers. ***B***, IHCs: presynaptic and postsynaptic immunolabels. CtBP2 (red) and GluA2 (green) immunopuncta were consistently juxtaposed at the IHCs. Magnified insert in the *x*- to *y*-plane shows clear separation of presynaptic and postsynaptic labels. The *x*- to *z*-labels and z- to y-labels were not as well segregated as those in OHCs. ***C***, Quantification of the number and the percentage of juxtaposed CTBP2 and GluA2 puncta in OHCs. ***D***, Quantification of the number and the percentage of juxtaposed CtBP2 and GluA2 puncta in the IHCs. *n* = 3-9 independent preparations; 50 IHCs, 72 OHCs for ***A–D***. There were no statistically significant differences in number or correlation among the immunolabels (one-way ANOVA test, *p* > 0.05). Scale bars: ***A***, ***B***, 5 µm; magnified inserts, 2.5 µm.

In initial experiments, antibodies specific to each of the AMPAR subunits, GluA1-4, as well as that to the GluA2/3 combination were applied to excised adult rat cochlear whole mounts (upper apical to middle turns). Among these, only anti-GluA2 produced localized punctate labeling below OHCs in the rat cochlea. A monoclonal mouse antibody and a polyclonal rabbit antibody provided comparable results, and so the resulting data were pooled for analysis and interpretation (see Materials and Methods). Double labeling with an antibody against CtBP2/RIBEYE (Wagner, 1997; Schmitz et al., 2000; Lenzi and von Gersdorff, 2001; Zenisek et al., 2003) was performed to relate postsynaptic GluA2 labeling to the location of presynaptic ribbons in hair cells (Fig. 1). With this combined labeling, both OHC and IHC afferent synapses were investigated in the organs of Corti of adult rats (2 months of age and older). The total number of puncta labeled by synaptic markers was counted in each *z*-stack and was divided by the number of hair cells. Hair cells were enumerated separately by background fluorescence of cell bodies at high light intensity, or by labeling of their nuclei with the CtBP2/RIBEYE antibodies.

The average number of GluA2 puncta per OHC was 2.3 ± 0.2, and the number of CtBP2 puncta per OHC was 2.4 ± 0.1 (*n* = 72 OHCs analyzed from three experiments; Fig. 1*C*). CtBP2 and GluA2 puncta were closely aligned in most cases (Fig. 1*A*, magnified inset). A juxtaposed CtBP2 punctum was present at 94.9 ± 1.1% of GluA2 puncta, and 85.5 ± 5.5% of CtBP2 puncta had an associated GluA2 punctum (Fig. 1*C*). Assuming that the GluA2 puncta represent functional synapses, this high level of juxtaposition suggests that AMPAR-mediated synaptic transmission occurs at ribbon synapses of OHCs, with two to three such ribbon synapses per OHC. The relationship of the GluA2 and CtBP2 immunolabels was examined further by rotating the original *z*-axis confocal stack into the *z*–*x*- and *z*–*y*-planes (Fig. 1*A*). From these viewpoints, the separation of red (CtBP2/RIBEYE) and green (GluA2) was better resolved.

In keeping with their known synaptic organization (middle cochlear turn), many more presynaptic ribbons and postsynaptic GluA2 receptor clusters were found among IHCs (Fig. 1*B*). At individual IHCs, there were 23.3 ± 0.6 GluA2 puncta and 22.4 ± 1.0 CtBP2 puncta (*n* = 50 IHCs from nine experiments; Fig. 1*D*). CtBP2-labeled ribbons and GluA2 puncta were consistently juxtaposed (Fig. 1*B*, magnified inset). For 96.8 ± 1.0% of CtBP2 puncta, a juxtaposed GluA2 punctum was found, and for 96.6 ± 1.1% of GluA2 puncta, a juxtaposed CtBP2 punctum was found (Fig. 1*D*). These results with GluA2 labeling echo previous reports regarding the number of synapses per IHC, and the close correspondence between CtBP2-labeled ribbons and GluA2/3 or GluA2 receptor clusters (Beutner and Moser, 2001; Brandt et al., 2003; Fuchs et al., 2003; Khimich et al., 2005; Neef et al., 2007; Meyer et al., 2009; Liberman et al., 2011). The separation of presynaptic CtBP2 and postsynaptic GluA2 puncta was better resolved in *z*-stacks of the IHCs than in those of the OHCs. This is probably due to a more horizontal disposition of IHCs in cochlear whole mounts, so synaptic labeling was viewed with the higher resolution of the *x*–*y*-image plane. This is in contrast to vertically oriented OHCs where presynaptic and postsynaptic elements appear to overlap in the *z*-axis, but could be better separated in the *x*- or *y*-axis.

**Figure 2.**
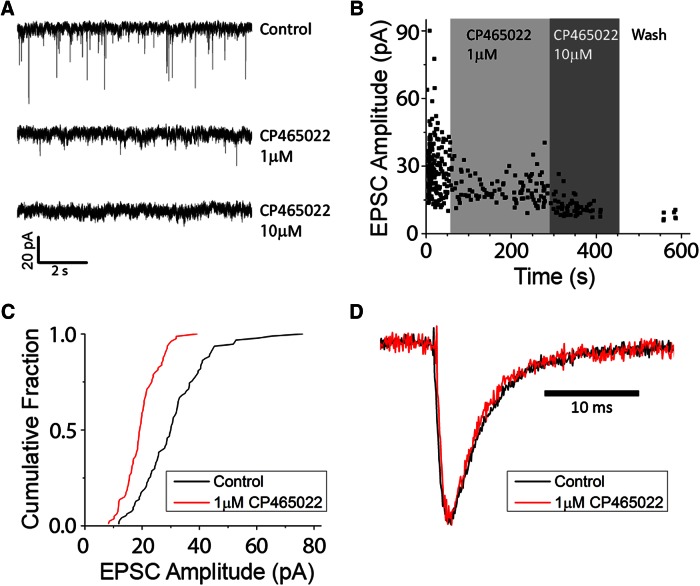
AMPA receptors mediate synaptic transmission from OHC to type II afferents in young (1- to 2-week-old) rat cochlea. ***A***, Inward synaptic currents (small downward deflections) evoked by high-potassium saline solution were reduced, then eliminated by exposure to CP-465,022. ***B***, Diary plot showing a partial block of EPSCs by 1 µm CP465,022 followed by complete block by 10 µm, then partial recovery after washout. Recordings [holding potential (Vhold), −80 mV] were made in 40 mm external potassium to increase EPSC frequency. ***C***, Cumulative fraction plot of EPSCs from fiber in ***B***. EPSC amplitudes decreased in the presence of 1 µm CP465,022 (red). ***D***, Scaled EPSC waveforms before (black) and during exposure to 1 µm CP465,022 (red) showing identical kinetics.

The identity of neurotransmitters and receptors at the OHC-to-type II afferent contact has been debated for some years. The absence of GluA2/3 immunoreactivity led to the logical conclusion that some mechanism other than AMPAR-mediated transmission operated there (Matsubara et al., 1996; Thiers et al., 2008). Initial studies of synaptic currents in type II afferents showed that these were blocked by the nonselective AMPA/kainate antagonist NBQX (Weisz et al., 2009), leaving open the possibility that postsynaptic kainate receptors respond to glutamate release from OHCs (Fujikawa et al., 2014). Further support for the involvement of GluA2-containing AMPA receptors was obtained by intracellular recording from type II afferents in excised apical turns of young rat cochleas (P9). The highly potent AMPA-specific antagonist CP-465,022 (Lazzaro et al., 2002; Balannik et al., 2005) was applied while recording potassium-evoked EPSCs (Fig. 2*A*). At 10 µm (three fibers) and 100 µm (two fibers), CP-465,022 completely eliminated EPSCs. Successive application of 1 µm then 10 µm CP-465,022 reduced EPSC amplitudes (Fig. 2*A*,*B*) going from partial to complete block, which is consistent with the reported potency of the drug on AMPA receptors (Lazzaro et al., 2002). At a concentration of 1 µm, CP-465,022 reduced the average EPSC amplitude by ∼50% (Fig. 2*C*). To further probe for kainate or other non-AMPA receptors, the amplitude of the residual current in CP-465,022 was normalized to the control amplitude, and its waveform was compared to that before the block. There was no difference in waveform before and during the block by CP-465,022 (Fig. 2D), although kainate receptor-mediated EPSCs have much slower kinetics than those served by AMPA receptors at CNS synapses (Lerma and Marques, 2013). Thus, if CP-resistant kainate receptors do contribute to synaptic currents in type II afferents, they were indistinguishable by kinetics or sensitivity to CP-465,022.

**Figure 3.**
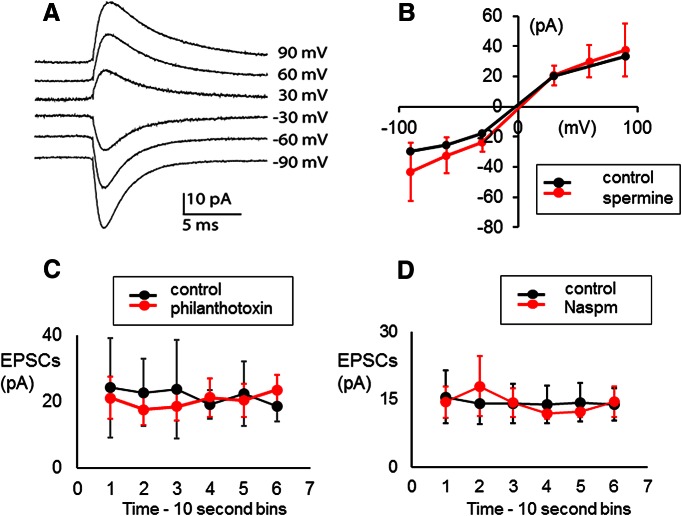
Calcium-impermeant glutamate receptors carry synaptic currents in type II afferents in young (1- to 2-week-old) rat cochlea. ***A***, Averaged synaptic currents in type II fiber containing 100 µm spermine at indicated membrane potentials (not corrected for junction potential). The number of events in each average current range from 115 to 624. ***B***, Current–voltage curve of synaptic currents for spermine-loaded type II fiber (red) compared with control data (black; panel is from the study by Weisz et al., 2009, used with permission). Average current amplitude with SDs shown for spermine data. ***C***, Average synaptic currents in a type II fiber (averaged over 10 s bins) before (black) and during exposure to 20 µm philanthotoxin (mean with SD). ***D***, Average synaptic currents in a type II fiber (averaged over 10 s bins) before (black) and during exposure to 10 µm Naspm (mean with SD).

Inclusion of the GluA2 subunit renders AMPARs impermeable to calcium (Hollmann et al., 1991; Mishina et al., 1991), requiring that synaptic currents in type II afferents should flow through calcium-impermeant channels if mediated by GluA2-containing AMPARs. This suggestion can be tested by examining the effects of intracellular spermine. This polyamine generates a voltage-dependent block of calcium-permeant glutamate receptors (i.e. non-GluA2 containing), resulting in a sharply rectified current–voltage relation (Donevan and Rogawski, 1995). So, the absence of rectification with intracellular spermine indicates the presence of calcium-impermeant GluA2 subunits. The voltage dependence of EPSCs in six type II fibers treated with intracellular spermine (100 µm) did not differ from control fibers (Fig. 3*A*,*B*, exemplar), which is consistent with low calcium permeability and the presence of GluA2 subunits. EPSCs in type II fibers also were unaffected by the compounds philanthotoxin (Tóth and McBain, 1998) and Naspm (1-naphthyl acetyl spermine; Tsubokawa et al., 1995), which act as channel blockers of non-GluA2-containing receptors. Neither philanthotoxin (20 µm, five fibers) nor Naspm (10 µm, five fibers) altered the average amplitude of EPSCs in type II fibers (Fig. 3*C*,*D*, exemplars).

Together with specific GluA2 immunolabeling in adult tissue (Fig. 1), these indicators and the sensitivity to CP-465,022 support the conclusion that, as for the IHC to type I synapse, GluA2-containing AMPARs mediate rapid glutamatergic excitation at the OHC-to-type II afferent synapse in the rat cochlea. If other receptor types participate, their involvement is indistinguishable from that of AMPARs in these recordings.

### Relationship of presynaptic ribbons and postsynaptic density proteins at IHC and OHC afferent contacts

Further insight into the synaptic arrangements of type I and type II afferent neurons was gained using antibodies directed against postsynaptic density proteins PSD-95, Shank, and Homer. These antibodies were applied to cochlear whole mounts, and their labeling was compared to that of GluA2 clusters and presynaptic ribbons at both inner and outer hair cell afferent contacts.

In the CNS, PSD-95 participates in the synaptic targeting of AMPA receptors through the coupling protein Stargazin and related transmembrane AMPA receptor regulatory proteins (Naisbitt et al., 1997; Sheng, 1997; Colledge et al., 2000; El-Husseini et al., 2000; Hirbec et al., 2003; Ives et al., 2004; Harms and Craig, 2005). PSD-95 binding partners also include NMDA receptors (Boeckers, 2006). In contrast to the near-membrane location of PSD-95, the postsynaptic density organizing protein Shank extends further into the cytoplasm to link glutamate receptor activity and local cytoskeletal remodeling, particularly within actin-rich dendritic spines (Brandstätter et al., 2004). Shank also interacts with mGluRs through the connecting protein Homer. The distribution of Shank and Homer was compared with that of PSD-95 and to the presynaptic ribbon marker CtBP2 at afferent contacts on IHCs in separate experiments.

**Figure 4.**
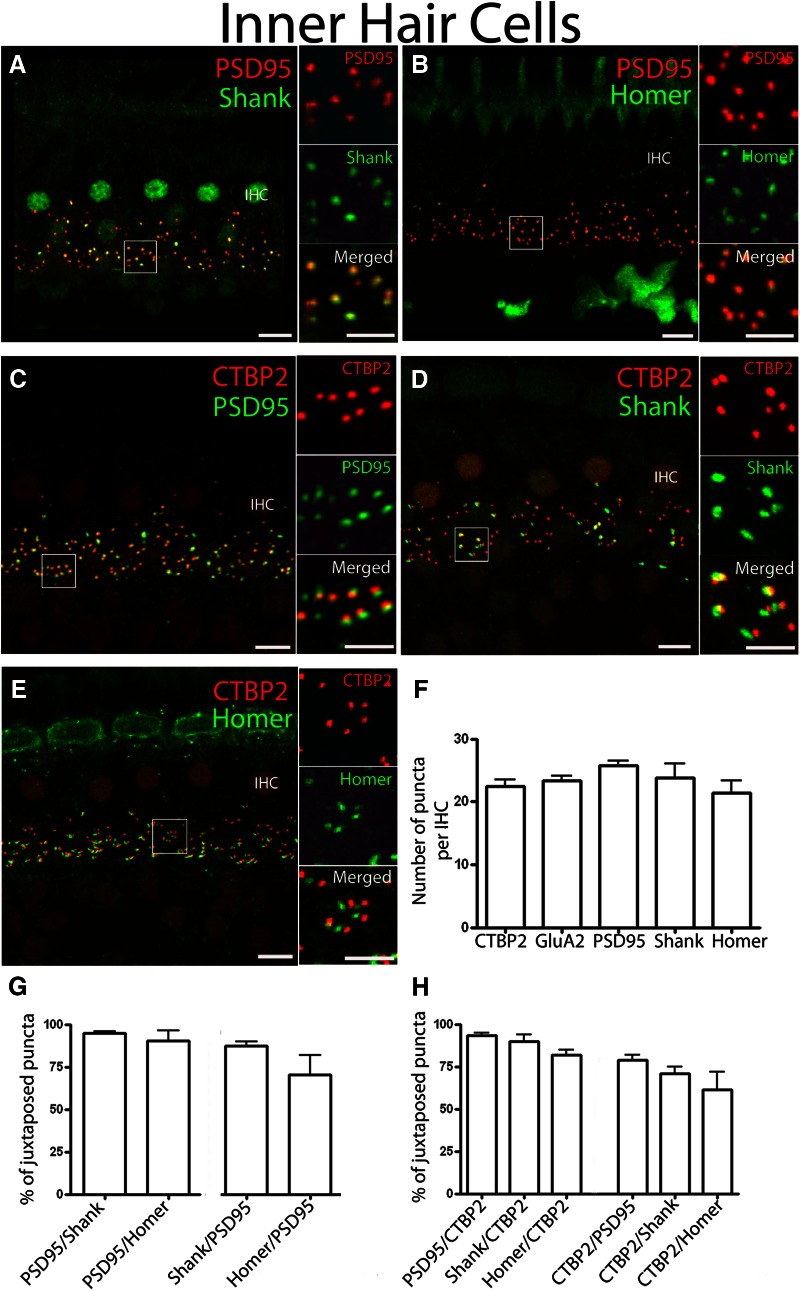
IHC synaptic immunopuncta. Confocal *z*-stacks of five IHCs in the middle turn of the organ of Corti from an adult rat viewed from the endolymphatic surface. ***A***, ***B***, Immunolabeling for postsynaptic density proteins PSD-95 (red channel) and Shank (green channel; ***A***) or Homer (green channel; ***B***) show closely coincident puncta of these postsynaptic density markers (magnified inserts in the *x*- to *y*-plane). ***C***, Immunolabeling for the CtBP2 red channel, and the postsynaptic marker PSD-95. Magnified insert shows juxtaposition in the *x*- to *y*-plane. ***D***, Immunolabel comparing CtBP2 and Shank distribution. ***E***, Immunolabel comparing CtBP2 and Homer. ***F***, Quantification of presynaptic and postsynaptic immunopuncta in IHCs. ***G***, Quantification of percentage juxtaposition of postsynaptic density proteins. ***H***, Quantification of percentage juxtaposition for presynaptic ribbon marker CtBP2 and the postsynaptic density proteins (PSD-95, Shank, and Homer). ***A–H***: *n* = 40-60 IHCs from four to five independent preparations. There were no statistically significant differences in number or correlation among these immunopuncta (one way-ANOVA test; *p* > 0.05). Scale bars: low magnification, 5 µm; high magnification, 2.5 µm.

The number of PSD-95 puncta per IHC in the adult rat cochlea was 25.8 ± 0.7, and the number of CtBP2/RIBEYE puncta was 22.4 ± 1.0 in double-labeling experiments (*n* = 60 IHCs in five mid-turn cochlear coils; Fig. 4*C*,*F*). Both Shank and Homer antibodies labeled type I boutons beneath IHCs in a pattern that closely corresponded with PSD-95 immunoreactivity (Fig. 4*A*,*B*). When comparing the number of puncta per IHC for CtBP2/RIBEYE, GluA2, PSD-95, Shank, and Homer, no significant differences were found (one way-ANOVA, *p* = 0.117), with all markers providing 21–26 puncta/IHC; PSD-95 provided the most, and Homer provided the least (Fig. 4*F*). For >90% of Shank puncta (94.6 ± 1.1%; seven cochlear segments) and Homer puncta (90.4 ± 5.8%; three cochlear segments), PSD-95 was located within 0.5 µm, suggesting that Homer and Shank are consistently expressed at postsynaptic densities of type I afferents (Fig. 4*G*). Likewise, Shank was largely associated with PSD-95 (Shank/PSD-95, 87.4 ± 2.4%). The difference in the numbers per hair cell may help explain the observation that most Homer puncta were juxtaposed with PSD-95; however, some PSD-95 puncta did not appear to be juxtaposed to Homer (70.5 ± 11.2% of Homer/PSD-95; Fig. 4*G*). This may reflect a lower signal-to-noise ratio for the Homer immunolabel or may suggest a real difference in expression among type I boutons.

Presynaptic ribbons labeled with CtBP2/RIBEYE antibodies were almost always juxtaposed with postsynaptic density proteins (Fig. 4*H*, three histogram bars on the left; PSD-95/CTBP2: 93.1 ± 1.6%; 10 cochlear segments; Shank/CtBP2: 89.9 ± 4.0%; 3 cochlear segments; Homer/CtBP2: 81.9 ± 2.7; 5 cochlear segments). Although not statistically significant, and so to be interpreted cautiously, the data might suggest that a minority of postsynaptic density puncta were not within the 0.5 µm surface-to-surface distance of CtBP2-immunolabled ribbons that was the criterion for juxtaposition. This fraction was larger for Homer than for Shank and for PSD-95, respectively (Fig. 4*H*, 3 histogram bars on the right; CTBP2/PSD-95, 78.9 ± 3.1%; CtBP2/Shank, 70.8 ± 4.3%; CtBP2/Homer: 61.3 ± 10.6%). The average surface-to-surface distance between CtBP2/RIBEYE puncta and Homer puncta was larger (0.05 ± 0.01 µm) than that for Shank (0.03 ± 0.01 µm) or PSD-95 (0.03 ± 0.01 µm), although it was not statistically significant (one way-ANOVA, *p* > 0.05). The significant observation is that every IHC had similar numbers of all immunopuncta. Thus, a majority of afferent contacts on IHCs included the presynaptic ribbon, postsynaptic density proteins, and GluA2-containing AMPARs. This was not the case for OHCs.

### Postsynaptic density proteins at OHC afferent contacts

**Figure 5.**
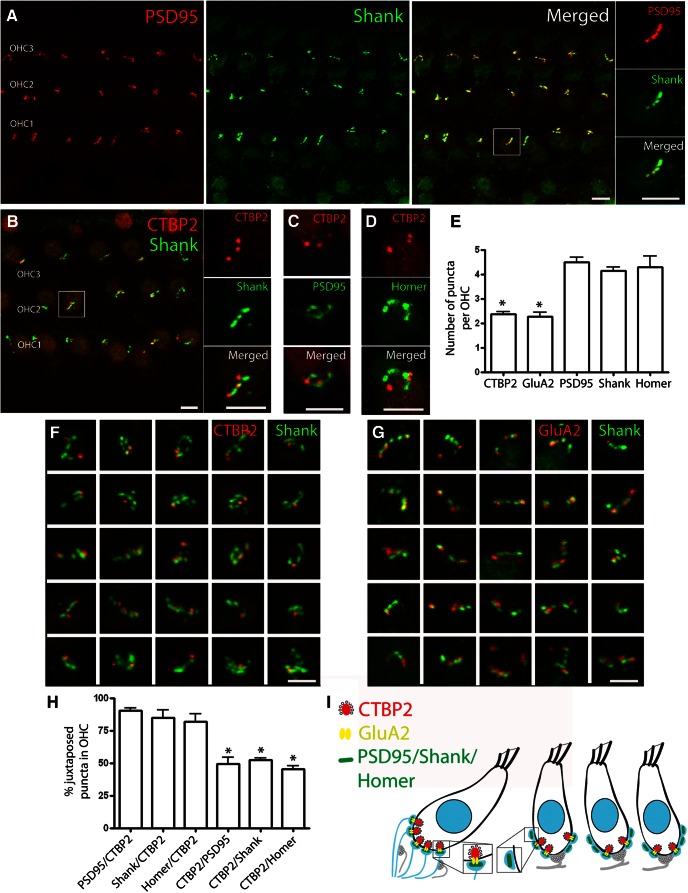
OHC synaptic immunopuncta. Confocal *z*-stack of OHCs in the middle turn of the organ of Corti from an adult rat viewed from the endolymphatic surface. ***A***, Immunolabeling with the postsynaptic density proteins PSD-95 (red channel) and Shank (green channel) show an interconnected series of puncta along the base of the OHCs. PSD-95 and Shank puncta are closely coincident (magnified insert, *x*- to *y*-plane). ***B***, Immunolabeling with the presynaptic ribbon marker CtBP2 (red) and postsynaptic marker Shank (green). ***C***, Immunolabel for CtBP2 (red) and PSD-95 (green). ***D***, Immunolabel for CtPB2 (red) and Homer (green). Magnified inserts (*x*- to *y*-plane) in each case show more extensive postsynaptic density distribution than presynaptic ribbon label. ***E***, Presynaptic and postsynaptic immunopuncta at the OHCs. There were significantly fewer CtBP2 or GluA2 puncta than postsynaptic density puncta (PSD-95, Shank, or Homer; one way-ANOVA, *p* = 0.01; Bonferroni’s multiple comparison test; *n* = 3-7; 72-168 OHCs). ***F***, Thumbnails of the base of individual OHCs immunolabeled for CtBP2 (red channel) and Shank (green channel). Many Shank immunopuncta had no associated CtBP2 puncta. ***G***, Thumbnails of the base of individual OHCs immunolabeled for GluA2 (red channel) and Shank (green channel). Many of the Shank immunopuncta had no associated GluA2 puncta. ***H***, Percentage juxtaposition of the CtBP2 and PSDs. The ratio of PSD-95, Shank, or Homer puncta juxtaposed to CTBP2 was significantly smaller than the ratio of CTBP2 puncta juxtaposed to the postsynaptic density proteins (one way-ANOVA, *p* ≤ 0.01; Bonferroni’s multiple comparison test; i.e., many PSDs had no ribbon). Scale bars: wide view, 5 µm; magnified inserts and thumbnails, 2.5 µm. ***I***, Schematic drawing of OHC and IHC synapses. At the IHC afferent synapse, CtBP2/GluA2 relates closely in number to postsynaptic density markers. At the OHC afferent synapse, only a subset of postsynaptic density proteins relates to CtBP2/GluA2 synaptic markers.

Having established the presence of Shank and Homer immunolabels at IHC afferent contacts, the distribution of immunolabels with those same antibodies was examined at the OHC afferent contacts. In contrast to the individual discrete puncta observed beneath IHCs, PSD-95, Shank, and Homer revealed more complex patterns that could extend several micrometers along the synaptic pole of the OHC. These appeared as an irregular cluster or as an interconnected series, like a short pearl chain (Fig. 5*A–D*,*F*,*G*). Postsynaptic densities beneath OHCs identified by the PSD-95 immunolabel were also positive for Shank in double-label experiments (Fig. 5*A*, insets). Homer was not tested in a colabeling experiment with other postsynaptic density markers, but showed the same “pearl chain” pattern as did PSD-95 and Shank (Fig. 5*D*). The number of postsynaptic density protein puncta per outer hair cell (PSD-95, 4.5 ± 0.2; Shank, 4.2 ± 0.1; Homer, 4.3 ± 0.5) was nearly twice the number of CtBP2 (2.4 ± 0.1) or GluA2 puncta (2.3 ± 0.2; one way-ANOVA, *p =* 0.01; Bonferroni’s multiple comparison test; *n* = 72-168 OHCs in three to seven cochlear segments; Fig. 5*E*). This contrasts markedly with equal numbers of these components per each IHC.

The pattern of postsynaptic density markers had an interesting relationship to the CtBP2-labeled presynaptic ribbons, as shown here for Shank (Fig. 5*B*,*F*). Most CtBP2-positive ribbons were juxtaposed to Shank (84.9 ± 5.7%), PSD-95 (90.6 ± 1.9%), and Homer (81.9 ± 6.2%; Fig. 5*H*). However, approximately half of the postsynaptic densities were ribbonless, with no associated CtBP2 puncta. Only 52.1 ± 1.9% of the Shank puncta, 49.2 ± 5.3% of PSD-95 puncta, and 45.6 ± 2.3% of Homer puncta had juxtaposed CtBP2 puncta. The percentages of PSD-95, Shank, or Homer puncta juxtaposed to CTBP2 were significantly lower than the percentage of CTBP2 puncta juxtaposed to the postsynaptic density proteins (one way-ANOVA, *p* ≤ 0.01; Bonferroni’s multiple comparison test; Fig. 5*H*).

These statistics and the strong correspondence between GluA2 immunoclusters and ribbons labeled with anti-CtBP2 (Fig. 1) suggest that GluA2 clusters might show a similar relationship to postsynaptic density proteins as does CtBP2. Indeed, double-label experiments with anti-GluA2 and anti-Shank revealed only partial correspondence, as found for anti-CtBP2 and anti-Shank (Fig. 5*G*). Thus, the number of postsynaptic densities, as defined by PSD-95, Shank, and Homer immunolabeling, was twice that of the ribbon-associated clusters of GluA2 receptors in type II afferents. Most GluA2 puncta were juxtaposed to Shank puncta; however, close to the half of Shank puncta did not have a juxtaposed GluA2 label (Fig. 5*G*). In other words, about half of the type II postsynaptic contacts, as defined by a PSD-95, Shank, or Homer immunolabel, may be “empty slots,” unable to mediate rapid glutamatergic transmission (Fig. 5*I*) since they are associated with neither GluA2-containing AMPARs nor ribbons.

### Type II fibers form a stereotyped pattern of OHC innervation

**Figure 6.**
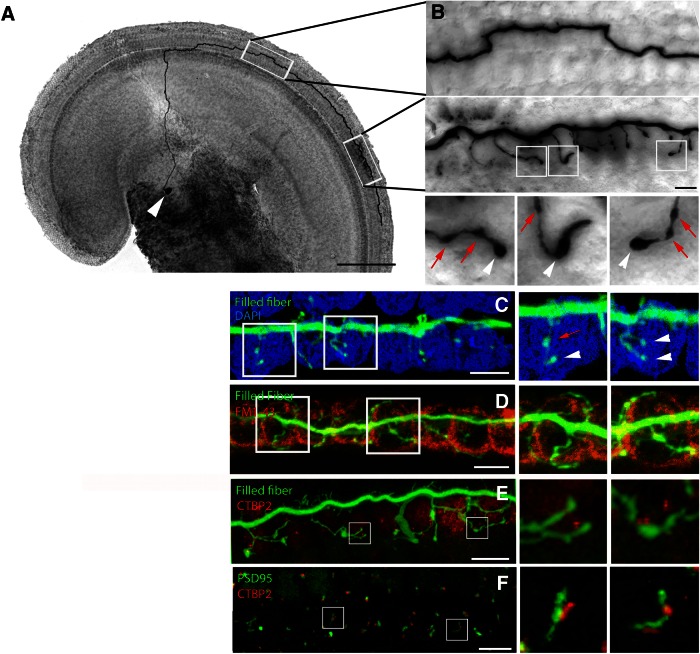
Single type II fibers visualized by intracellular labeling. ***A***, Apical turn of a young (P8) rat organ of Corti with biocytin-filled type II fiber after streptavidin–peroxidase reaction. Scale bar, 125 µm. ***B***, Higher magnification of boxed areas in ***A***, showing trajectory and terminal branches. ***C***, Biocytin-filled type II fiber reacted with streptavidin-Alexa Fluor 488 (green). OHC nuclei labeled with DAPI (blue). Magnifications show en passant (red arrows) and terminal (white arrowheads) swellings of branches from boxed regions. ***D***, Biocytin-streptavidin-Alexa Fluor 488-filled fiber combined with FM1-43-labeled OHCs (red). Magnifications show terminal branches enwrapping the base of outer hair cells. ***E***, Biocytin-streptavidin-Alexa Fluor 488-filled fiber combined with a CtPB2 immunolabel (red). Magnifications show an approximation of some terminal branches to CtBP2 puncta. ***F***, Combined immunolabel for PSD-95 (green) and CtBP2 (red) among OHCs of young rat cochlea. Magnification shows pearl chain pattern found in adult cochlea. Scale bars: ***B–G***, 5 µm; magnifications, 2.5 µm.

How do the pearl chain patterns of the PSD immunolabels relate to the terminal arbors of individual type II fibers? These extend spiral dendrites that contact numerous OHCs (Perkins and Morest, 1975; Ginzberg and Morest, 1983, 1984; Berglund and Ryugo, 1987; Brown, 1987; Simmons and Liberman, 1988a,b; Liberman et al., 1990; Echteler, 1992; Huang et al., 2007; Koundakjian et al., 2007). To label the peripheral type II fibers and to understand their specific connectivity with OHCs, a giga-ohm-seal intracellular recording was used to fill type II fibers under OHCs with biocytin in excised apical turns of cochleas from young rats (P7–P9). After streptavidin-peroxidase processing, 15 type II fibers were visualized and measured from their somata in the spiral ganglion (Fig. 6*A*, white arrowhead) to their basal-most endings along the cochlear spiral. The peripheral neurite leaves the soma in the spiral ganglion to cross the floor of the tunnel of Corti and turns ∼90° to travel toward the cochlear base along the outer spiral bundle (Fig. 6*A*), sometimes switching between OHC rows (Fig. 6*B*). Over half of the filled fibers (8 of 15 fibers) had a single spiral process that averaged 714 ± 81 µm (*n* = 8) from the turning point to the basal-most tip (Table 1). An average of 17 ± 1.4 short branches off the spiral process formed en passant (Fig. 6*B*,*C*, red arrows) and terminal (Fig. 6*B*,*C*, white arrowheads) swellings. These terminal branches tended to cluster (12 ± 1 branches; spanning a mean distance of 139 ± 19 µm; *n* = 8) with smaller secondary clusters, ≥100 µm distant in some cases. In one of the eight “single-process” fibers, two synaptic zones 228 µm apart had nearly equal branching (12 and 8 branches). The spiral dendrite also could split into two (6 of 15 fibers) or three (1 fiber) basally projecting processes (average length, 619 ± 79 µm). One such fiber branched as it crossed the tunnel of Corti, and one fiber had branches extending both basally and apically. Even including these exceptions, the overall length of the spiral process, and the number of synaptic branches and terminal arborization zones were similar among all 15 fibers (Table 1). The number of branches within the terminal arbors of all the type II fibers averaged 16 ± 1.4 (*n* = 15). These terminal branches had an average length of 10.9 ± 1.7 µm. Terminal branches had an average of 2.0 ± 0.2 en passant swellings in addition to the terminal bouton. Each terminal branch contacted one to three OHCs in the same row. The average total number of OHCs contacted by each type II fiber was 23.7 ± 1.5. Some branches showed arching shapes (Fig. 6*C*, inset), which could correspond to the pearl chain postsynaptic densities described in Figure 5. Although some fibers split into two or three, in five of seven such cases, terminal branches arose from only one of the arbors, or prior to the branch point, so that all 15 fibers, whether possessing one or more major processes, had similar numbers of terminal branches and, presumably, equivalent numbers of synaptic contacts.

**Table 1:** Morphology of biocytin-filled type II fibers in the apical turn of young rat cochleas

Fiber number	Spiral process (µm)				Major synaptic area		
		Distance to apex (µm)	Arbors (*n*)	Total synaptic branches	Distance from apex (µm)	Length (µm)	Branches (*n*)
1	899.2	843	1	21	1532	97.3	13
2	516.4	1121	1	13	1448.3	150.2	9
3	1194.7	566.5	1	21	1618.3	165.2	11
4	617.8	613.5	1	12	1082.2	160.5	12
5	585.5	997.8	1	15	1109	52.8	9
6	733.9	658.1	1	22	1108.3	161.3	12
7	549.1	608	1	17	920.3	224.8	17
8	612.8	Not available	1	13	Not available	101.3	12
damage							
Average ± SEM	714 ± 81	773 ± 77		17 ± 1.4	1260 ± 95	139 ± 19	12 ± 1
9	400.65	488.8	2	8	692	60.9	8
10	720.1	983	2	14	1243.4	168	11
11	983	577	2	7	1255.2	139.6	7
12	689.1	613	2	20	1095.4	135.2	15
13	413.5	479.5	3	24	700	130.245	18
14	475.5	919	2	13	1107.8	149.5	13
15	649.7	924	2	23	1423.4	193.4	20
Average ± SEM	619 ± 79	712 ± 83		16 ± 3	1074 ± 106	140 ± 15	13 ± 2
Total average ± SEM	669 ± 56	742 ± 55		16 ± 1	1167 ± 72	139 ± 12	12 ± 1

The location of the main terminal arbor (synaptic input zone) ranged from 700 to 1600 µm (average, 1167 ± 72 µm; *n* = 15) from the cochlear apex, placing the synaptic area in a frequency range of ∼9 kHz (Müller, 1991). On the other hand, the 90° turning point of the fibers was located at 500–1000 µm (average, 742 ± 55 µm; *n* = 15) from the apex, placing it in the frequency range of 7 kHz. Thus, as noted previously (Brown, 1987), type II afferents, if sufficiently sensitive, would report vibrations one-quarter to one-half octave higher in frequency than type I afferents projecting in parallel to the same tonotopic zone of the cochlear nucleus.

The number of fibers per OHC in the biocytin/streptavidin/peroxidase labeling was determined by identifying labeled branches with a bright-field microscope, and counting individual OHCs by shape and location. To verify these results, fluorescent labeling of the fiber (biocytin, Streptavidin-Alexa Fluor 488) was combined with fluorescent labeling of OHCs. In one set of experiments, OHC nuclei were counterstained with DAPI (Fig. 6*C*). In a second set of experiments, the tissue was incubated with 5 µm FM1-43, a fluorescent dye that is taken up by hair cells through the transduction channel (Nishikawa and Sasaki, 1996; Fig. 6*D*), for 30 s. The main terminal zone of a filled fiber was investigated with confocal microscopy. Again, branching fibers and fibers with bouton endings and *en passant* swellings were visible, and branches appeared to arc around the synaptic pole of the OHC (Fig. 6*D*, insets). In this dataset, the number of OHCs contacted by one fiber was 23 ± 2.2 (*n* = 9), which is identical to the result in preparations with unlabeled OHCs.

The combination of a presynaptic or postsynaptic immunolabel with fiber filling was only occasionally successful. This may be a result of tissue condition after the time required for intracellular recording, and/or a reflection of less robust expression of synaptic proteins in the 8- to 10-d-old animals needed for successful fiber recording. In any event, even this low success rate provides qualitative, if not quantitative, description. CtBP2 immunopuncta were located near to some, but not all, terminal swellings of a filled fiber (Fig. 6*E*), reinforcing the possibility that type II fibers can form nonfunctional contacts with OHCs. Additional immunolabeling was performed on young cochlear whole mounts that were processed in a manner similar to that of the adult tissues. Double immunolabeling for CtBP2 and PSD-95 gave an intermittent pearl chain association like that onto adult OHCs (Fig. 6*F*).

## Discussion

Our understanding of synaptic transmission between hair cells and afferent neurons of the cochlea has advanced gradually (Guth et al., 1976; Hudspeth, 1997; Fuchs et al., 2003; Fuchs, 2005; Ruel et al., 2007; Glowatzki et al., 2008; Meyer and Moser, 2010; Defourny et al., 2011). Neuroanatomical studies led the way, with the description of two distinct classes of neurons that differentially innervate inner and outer hair cells, with type I afferents making single contacts with single IHCs and type II afferents contacting ≥5-28 OHCs (Perkins and Morest, 1975; Spoendlin, 1975; Pujol et al., 1978; Ota and Kimura, 1980; Kiang et al., 1982; Ruggero et al., 1982; Nadol, 1988). Glutamatergic transmission from inner hair cells to type I afferents has been well accepted for many years (Guth and Bobbin, 1971; Guth et al., 1976; Bledsoe et al., 1981; Pujol et al., 1985; Bobbin et al., 1991; Ottersen et al., 1998; Glowatzki and Fuchs, 2002), but the same was firmly established for outer hair cells only with the advent of intracellular recordings from type II fibers (Weisz et al., 2009, 2012).

A remaining unknown element was the identity of the glutamatergic receptor in type II afferents. Antibodies to the GluA2/GluA3 AMPA receptor heteromer or to the GluA2 subunit reliably labeled type I afferent contacts beneath inner hair cells, but failed to do so at the adult OHC–type II connection in earlier studies (Matsubara et al., 1996; Knipper et al., 1997; Eybalin et al., 2004; Meyer et al., 2009; Liberman et al., 2011; Fujikawa et al., 2014). However, the present work shows that GluA2-specific antibodies labeled postsynaptic receptor clusters beneath both inner and outer hair cells of adult rats. Two different GluA2 antibodies gave the same result and failed to label contacts onto IHCs of GluA2-null mice. GluA2 receptors were found in postsynaptic densities that colocalized with presynaptic ribbons in OHCs, but were absent from postsynaptic densities that did not face presynaptic ribbons. Intracellular recordings from type II afferents showed that synaptic currents were sensitive to the AMPA-selective antagonist CP-465,022 and were carried by calcium-impermeant channels, which is consistent with inclusion of the GluA2 subunit. Thus, biophysics, pharmacology, and immunohistology support the conclusion that AMPA-type receptors (GluA2 containing) mediate glutamatergic transmission from both IHCs and OHCs onto their respective afferents. While electrophysiological evidence comes only from apical segments of the young rat, immunolabeling for GluA2 below OHCs was found in middle turns of adult rat cochleas, supporting the conclusion that AMPA receptors serve this synapse throughout life. The presence of GluA2 does not rule out the participation of other components, such as kainate receptors (Peppi et al., 2012; Fujikawa et al., 2014), but their contribution is either undetectable in these recordings or indistinguishable from that of AMPA receptors. It will be of interest to determine whether more subtle modulatory effects might depend on kainate receptor activity.

Type I and type II afferents differ in morphology, cochlear innervation pattern, synaptic transfer function, and resistance to acoustic trauma. These differences might be reflected in, or even dependent upon, the molecular composition of their synaptic contacts. GluA2 immunolabeling seemed generally fainter in type II than type I dendrites, perhaps indicating a lower density of receptors in each cluster. However, the chief distinction was that the postsynaptic densities of type II afferents (defined by the immunopuncta of any of the PSD-95, Shank, or Homer afferents) are more numerous than the GluA2 clusters immediately opposite synaptic ribbons of the outer hair cell; in contrast to the equal numbers of receptor clusters, ribbons and postsynaptic densities at the IHC to type I contacts. This difference may reflect in part the structure of the afferent ending itself. Type I afferents terminate in single small, unbranched boutons opposite IHC ribbons. In contrast, type II afferents extend hundreds of micrometers along the outer hair cell rows and form functional synapses with at least 10, and probably many more, OHCs (Perkins and Morest, 1975; Ginzberg and Morest, 1983; Berglund and Ryugo, 1987; Brown, 1987; Simmons and Liberman, 1988a; Fechner et al., 2001; Jagger and Housley, 2003; Nayagam et al., 2011; Weisz et al., 2012). The area of contact with the OHC has been described as a discrete bouton in some studies (Nadol, 1983; Ginzberg and Morest, 1984) but can be more extensive, forming en passant synapses as it travels past the OHC (Nadol, 1988; Francis and Nadol, 1993; Sobkowicz et al., 1993). The pearl chain pattern observed here with PSD-95, Shank, or Homer immunolabeling is consistent with this description of *en passant* as well as terminal contacts with OHCs and corresponds with the “C-shape” pattern described previously (Fujikawa et al., 2014). GluA2 immunopuncta align with only a subset of postsynaptic densities in a pearl chain, but are closely correspondent with presynaptic ribbons. Thus, some postsynaptic densities of type II neurons appear to be empty slots, without GluA2 receptors and lacking presynaptic ribbons.

We conclude that AMPA-mediated synaptic currents result from vesicular glutamate released at ribbons facing GluA2-positive terminals of type II afferents. This leaves unresolved the question of what, if any, transmission also might occur at ribbonless contacts. Kainate receptors are found at ribbonless contacts of type II afferents onto rat OHCs (Fujikawa et al., 2014), but it remains to be determined what role they play. NMDA receptors have been implicated in the plasticity of afferent contacts on IHCs (Usami et al., 1995; d'Aldin et al., 1997; Knipper et al., 1997; Chen et al., 2009), but there is no evidence as yet for a role in type II afferents. Finally, Homer could provide an anchor for metabotropic glutamate receptors in cochlear afferents (Safieddine and Eybalin, 1995; Niedzielski et al., 1997; Kleinlogel et al., 1999; Peng et al., 2004). An experimental design that reveals longer-lasting, modulatory changes in excitability may be required to directly assess putative non-AMPAR inputs.

An intriguing proposition is that ribbonless contacts provide a reservoir of plasticity for the type II afferents, somewhat like the silent synapses found in the CNS (Kerchner and Nicoll, 2008), although requiring both presynaptic ribbons and postsynaptic AMPARs for activation. Long-term plasticity in the hippocampus results in part from the insertion of AMPA receptors into the postsynaptic density of previously silent synapses (Naisbitt et al., 1997; Sheng, 1997; Petralia et al., 1999; Colledge et al., 2000; El-Husseini et al., 2000; Liao et al., 2001; Hirbec et al., 2003; Ives et al., 2004; Harms and Craig, 2005). Several observations suggest that type II afferents may be able to adapt to changing cochlear conditions. First, type II afferents are resistant to cochlear trauma, remaining even after OHC damage (Spoendlin, 1971; Ryan et al., 1980; Kujawa and Liberman, 2009). Second, “empty” PSDs could provide a substrate for enhanced transmission by the addition of GluA2 receptors (and presynaptic ribbons in the hair cell). Perhaps previously silent synapses “awaken” to replace lost inputs, especially given the extensive arbors of type II afferents that could span the boundaries of damaged regions. Third, the numbers of afferent and efferent synapses are reciprocally related during the postnatal maturation of IHCs (Katz et al., 2004; Roux et al., 2011; Johnson et al., 2013), and efferent synapses return to the partially denervated IHCs of aged, deaf mice (Lauer et al., 2012; Zachary and Fuchs, 2015). Fourth, OHCs in mice with reduced or absent efferent function have more type II afferent contacts with ribbons than do wild-type OHCs (Pujol and Carlier, 1982; Fuchs et al., 2014), although other work failed to find this effect (Liberman et al., 2000). Type II afferents can be activated by cochlear tissue damage (Liu et al., 2015), which could serve as a trigger for activity-dependent enhancement of synaptic connectivity. It will be of interest to examine the distribution of synaptic proteins in type II afferents within, or spanning the boundaries of, regions of outer hair cell damage.

## Synthesis

The decision was a result of the Reviewing Editor Christian Hansel and the peer reviewers coming together and discussing their recommendations until a consensus was reached. A fact-based synthesis statement explaining their decision and outlining what is needed to prepare a revision is listed below.

The two previous expert reviewers at Journal of Neuroscience have clearly stated that the manuscript is well done and that the experiments are very good, too. Reviewer 2 points out that the paper largely confirms previous studies.

As eNeuro is less about novelty, but about scientific rigor and quality, the manuscript is ready for publication in eNeuro as is.
